# Effect of mobile text messages on antiretroviral medication adherence and patient retention in early HIV care: an open-label, randomized, single center study in south Florida

**DOI:** 10.1186/s12981-020-00275-2

**Published:** 2020-05-13

**Authors:** Elizabeth M. Sherman, Jianli Niu, Shara Elrod, Kevin A. Clauson, Fadi Alkhateeb, Paula Eckardt

**Affiliations:** 1 Department of Pharmacy Practice, Nova Southeastern University College of Pharmacy, 3200 South University Drive, Fort Lauderdale, FL 33328 USA; 2 Division of Infectious Diseases, Memorial Healthcare System, 5647 Hollywood Boulevard, Hollywood, FL 33021 USA; 3 Office of Human Research, Memorial Healthcare System, 3111 Stirling Road, Hollywood, FL 33312 USA; 4 Department of Pharmacotherapy, University of North Texas System College of Pharmacy, 3500 Camp Bowie Boulevard, Fort Worth, TX 76107 USA; 5 Department of Pharmacy Practice, Lipscomb University College of Pharmacy, One University Park Drive, Nashville, TN 37204 USA; 6 Qatar University College of Pharmacy, QU Health, Building Ibn Al-Bitar (I06), Doha, Qatar

**Keywords:** HIV, Medication adherence, Text message, SMS, Mobile health

## Abstract

**Background:**

People with HIV (PHIV) with limited access to health services often experience suboptimal antiretroviral therapy (ART) adherence. We investigated whether a daily text messaging intervention improves ART adherence and retention in early HIV care in PHIV in a south Florida hospital-based clinic.

**Methods:**

ART-naïve PHIV receiving care through the clinic’s Ryan White HIV/AIDS Program were enrolled and randomly assigned to the intervention or control groups with a 1:1 ratio. The intervention group received a 1-way text message daily and the control group received standard care without receiving text message reminders for 6 months. HIV RNA and CD4 cell count were measured at baseline and post-intervention. Adherence to ART was defined as a visual analog scale of ≥ 90%. Retention in care was defined as continued engagement at study end.

**Results:**

94 ART-naïve patients were randomized and 83 (85.6%) completed the study, of which 44 were in the intervention group and 39 were in the control group. At the end of the 6-month study period, adherence to ART was 84.4% in the intervention group versus 73.5% in the control group (OR, 1.9; 95% CI 0.7–5.0; *p* = 0.194). Retention in care significantly improved in the intervention group compared to the control group with the odds of retention increasing by 20% (OR, 1.2; 95% CI 1.1–1.5; *p* = 0.006). Undetectable HIV RNA (< 50 copies/mL) was 86.7% in the intervention group versus 73.5% in the control group (OR, 2.3; 95% CI 0.8–6.9; *p* = 0.112). A significant increase in CD4 cell count and a decrease in HIV RNA were found at study end, with no differences between the two groups.

**Conclusions:**

In this pilot study, a one-way daily text messaging intervention did not improve ART adherence over a 6-month study period, but significantly enhanced patient retention in early HIV care. Implementation of interventions to improve adherence in this population is required.

## Introduction

Antiretroviral therapy (ART) has drastically decreased morbidity and mortality in people with human immunodeficiency virus (PWH) with high levels of adherence required to achieve long-term clinical benefit [1]. Poor ART adherence and lack of retention in medical care are associated with virologic failure, emergence of resistant viral strains, progression to advanced disease, and increased risk of HIV transmission to others [1, 2]. US President Donald J. Trump recently announced his administration’s goal to end the US HIV epidemic in the next 10 years through multi-modal pillars, including expansion of antiretroviral treatment to achieve sustained viral suppression [3].

Currently in the US, it is estimated that 86% of all PWH have been diagnosed, 49% are retained in care, and 53% have achieved viral suppression, the ultimate goal of ART [4]. The large number of PWH who are not retained in care and have not achieved viral suppression is concerning since it is estimated that 80% of new HIV transmissions are from persons who do not know they have HIV infection or are not retained in care [5]. ART adherence level varies depending on different population contexts [6–8]. There is emerging evidence that poor ART adherence is more common in socioeconomically disadvantaged, underserved, and/or less educated populations, as patients who lack medical/prescription insurance, or have policies with poor coverage, are less likely to see medical providers or fill prescriptions, due to inability to pay for services [9–11]. Therefore, it is vital to identify interventions improving ART adherence and retention in care in this population.

The Federal Ryan White HIV/AIDS Program (RWHAP) serves as a safety-net program, providing HIV-related health services to more than half a million low-income PWH without sufficient health coverage. In 2017, 85.9% of RWHAP patients were virally suppressed, exceeding the national average of 59.8% [12].

In recent years, short messaging service (SMS) “text messages” interventions have been implemented in HIV care in resource-limited settings with some reporting improved adherence and health outcomes [13–16] and some reporting no benefits [17, 18]. In the US, SMS reminders have been shown to promote ART adherence for adolescents and youth with HIV [19, 20]. A recent meta-analysis of all HIV adherence-related SMS studies revealed that these reminders are a promising intervention to increase HIV care adherence and further study should focus on which populations benefit the most from this intervention [14]. However, no validated data exist utilizing SMS reminders as a means of improving ART adherence in RWHAP adult patients.

The primary objective of this study was to determine whether a daily SMS reminder intervention improves ART adherence at the end of a 6 month study period when compared to standard of care in adult PWH initiating ART in a RWHAP-funded clinic. The key secondary objectives were to (1) determine whether a daily SMS reminder intervention improves engagement in clinic care at 6 months; and (2) determine whether a daily SMS reminder intervention improves virologic suppression and CD4 cell counts at 6 months post-intervention when compared to standard of care in this population.

## Methods

### Study population and setting

This was a randomized, parallel, single-center, controlled pilot study evaluating the impact of 1-way daily text message reminders on ART adherence and retention in care among PWH in a south Florida RWHAP clinic. Participants were identified by clinician referral and were screened for eligibility. Participants were included in the study if they met all of the following criteria: (1) older than 18 years of age and able to give informed consent; (2) documented HIV infection; (3) English literate; (4) own a cell phone with SMS capability; (5) HIV treatment naive, initiating ART for the first time or started ART within 1 month of eligibility screening. Individuals were excluded if they had any of the following: dementia, blindness or severely impaired vision uncorrectable with eyeglasses, or deafness or hearing problems uncorrectable with a hearing aid. These exclusion criteria mirror those utilized in the development and validation of the health literacy assessment tool employed in our study, which involves visual cues and required the ability to follow oral directions [21]. All eligible participants provided informed consent to participate in the study, which was approved by the institutional review boards of Nova Southeastern University, Memorial Healthcare System, and Florida Department of Health.

### Randomization and intervention

Recruitment began in September 2011 and ended in April 2014. Participants who signed informed consent were randomized to the intervention or control group in a 1:1 ratio using a computer-generated allocation sequence. Intervention group participants received the same standard of care as the control group plus received automated 1-way medication reminders (i.e., “Here’s to your health!”) delivered via SMS (Memotext, Toronto, Ontario, Canada). The Memotext platform was selected because it has been clinically and commercially validated to be an effective SMS intervention [22]. The “Here’s to your health!” message was the only message utilized and was developed using best available evidence and approved by clinic providers before implementation. Messages were delivered daily for 6 months, and message delivery was timed to coincide with individual medication usage schedule. Patients in the intervention arm were asked for a convenient time to receive the message based on the time of day they took their ART medication and messages were scheduled accordingly. Message receipt was confirmed for accuracy and timely delivery at study follow-up appointments. In the control group, participants received the standard of care, which included verbal reminders about their upcoming medical appointments and ART adherence at baseline and follow-up appointments, without receiving SMS reminders. All participants received standard face-to-face medication education including: (1) the dosing and timing of medications (with/without meals, time of day), (2) the importance of taking medication exactly as prescribed, (3) strategies for promoting successful adherence, (4) potential significant side effects, and (5) recommended actions for problems.

### Data collection and outcome measures

Participants’ demographic and clinical characteristics, and health literacy measured by the Rapid Estimate of Adult Literacy in Medicine-Short Form (REALM-SF) tool [21] were collected at the time of enrollment. CD4 cell count, obtained by flow cytometry, and HIV RNA, obtained by real-time PCR, were abstracted from the patient’s chart at baseline, 3- and 6-months post-intervention. The primary outcome of the study was ART adherence. A visual analogue scale (VAS) was used to measure self-reported ART adherence on a scale of 0–100, with 0 for taking no medication and 100 for taking all of the prescribed medication. The VAS instrument has been previously validated to accurately measure ART adherence [23]. Adherence was calculated as the percentage of pills self-reported as taken from the total number of prescribed pills over the past 30 days. Participants who reported an intake of ≥ 90% of the prescribed pills, a threshold level of adherence required for virologic suppression [24], were considered adherent, and those with a reported intake < 90% were classified as non-adherent. The proportion of participants with an undetectable HIV RNA (defined as < 50 copies/mL) was documented at the time of follow-up visits. Retention in care was calculated as the number of participants in care at the clinic at the time of follow-up visit divided by the total number of patients enrolled in the treatment arm.

### Statistical analysis

This was an unpowered pilot study. Continuous variables are presented as mean (range) and mean ± standard deviation (SD), and categorical variables as numbers and percentages. For comparison between groups, Chi-square test was used for categorical variables, while unpaired *t* test was used for continuous variables. All individuals lost to follow-up were classified as non-adherent for the study period. Primary outcome was analyzed by an intention-to-treat approach and in a per-protocol analysis, respectively. Per protocol analysis included data available on all randomized participants completing follow-up visits at 3 and 6 months post-intervention. Proportion of patients retained in care was estimated using the Kaplan–Meier method and a log-rank test was used to compare probability of retention in care between the two groups. All statistical analyses were performed using GraphPad Prism 7.0 (San Diego, CA), with a two-tailed p-value of < .05 considered statistically significant.

## Results

### Baseline characteristics of the study population

**Fig. 1:**
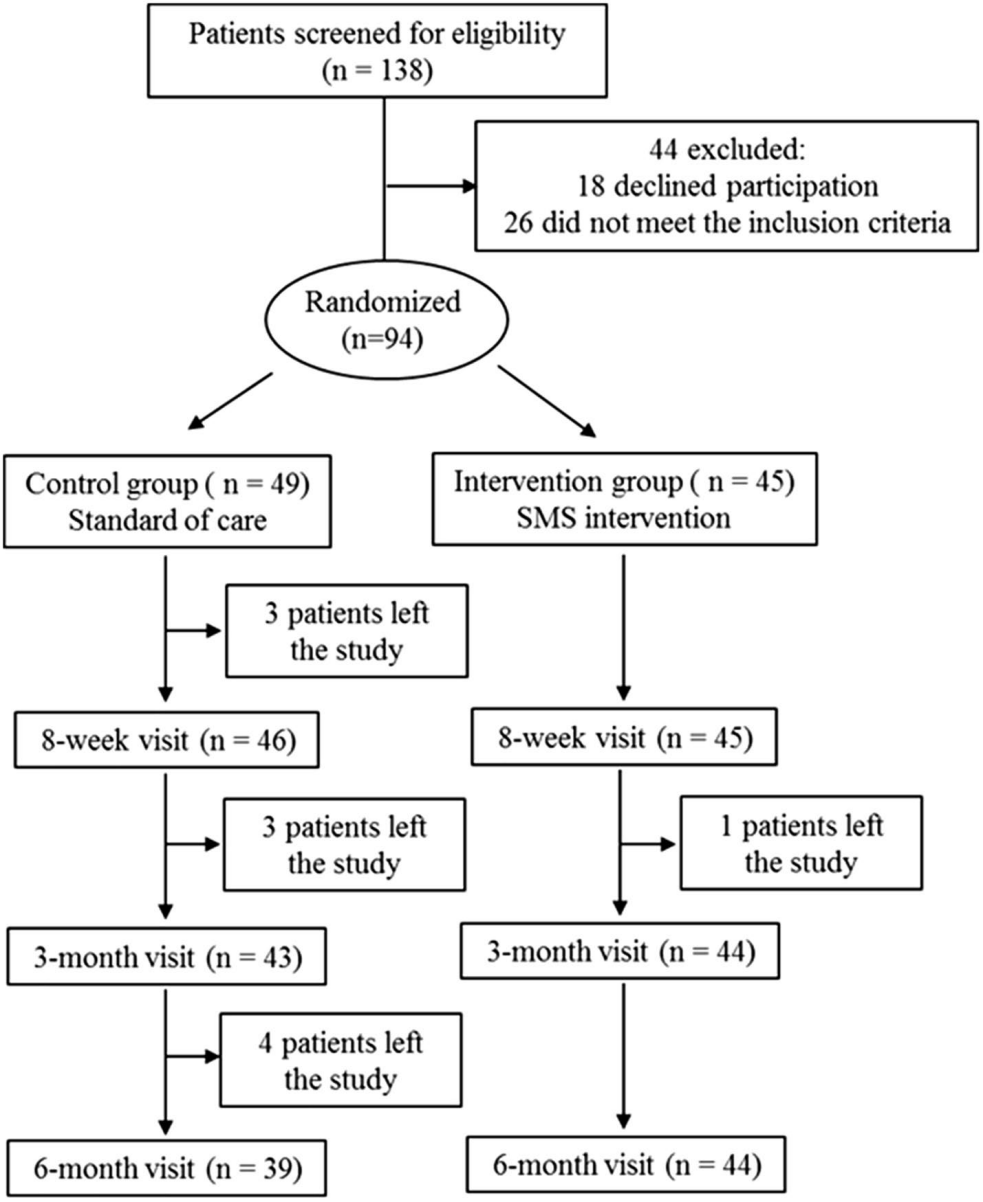
Study flow chart

Over a period of 31 months, 138 participants were screened for eligibility, of which 26 did not meet eligibility criteria and 18 declined participation. The remaining 94 participants were enrolled and randomly allocated to either the control (n = 49) or intervention group (n = 45). Figure 1 summarizes the recruitment and study flow of participants. Within the 6-month study intervention period, 10 participants left from the study (participants were either lost to follow-up or transferred care to a different clinic) in the control group and 1 left from the study in the intervention group. Hence, 94 patients (49 in the control group and 45 in the intervention group) were included in the intention-to-treat analysis, and 83 patients who completed the study per-protocol were included in the per-protocol analysis.

Table 1 summarizes demographics and clinic characteristics of participants at the time of enrollment. Mean age was 39.2 ± 11.3 years and 61.7% were male. A majority of the participants were black (63.8%) and 62.8% (59/94) reported heterosexual HIV transmission. Mean REALM-SF score was 6.1 ± 1.3, corresponding to a 7th to 8th grade literacy level. Both groups were well matched in their demographics, mode of HIV transmission, and baseline REALM-SF score. There were no significant differences in baseline clinical characteristics including HIV RNA, CD4 cell count, and ART regimen between the two groups (Table 1).

**Table 1:** Baseline demographic and clinic characteristics of study patients

	Control (n = 49)	Intervention (n = 45)	*p* value
Age, years			
Mean ± SD	40.7 ± 10.8	37.5 ± 11.7	0.171
Median (range)	42 (18–63)	36 (21–60)	0.128
Gender, n (%)			
Cisgender men	30 (61.3)	28 (62.2)	0.921
Cisgender women	18 (36.7)	17 (37.8)	0.917
Transgender women	1 (2)	0 (0)	0.335
Ethnicity, n (%)			
White	4 (8.2)	4 (8.9)	0.899
Black	28 (57.1)	32 (71.1)	0.159
Hispanic	16 (32.7)	9 (20)	0.165
Other	1 (2)	0 (0)	0.335
Mode of HIV transmission, n (%)			
Heterosexual	30 (61.3)	29 (64.4)	0.832
MSM	15 (30.6)	12 (26.7)	0.820
Other	3 (6.1)	4 (8.9)	0.706
Viral load, log_10_ copies/mL			
Mean ± SD	4.7 ± 0.8	4.7 ± 1.0	1.000
Median (range)	4.8 (2.4–6.9)	4.7 (2.2–6.8)	0.779
CD4 cell count, cells/mL			
Mean ± SD	221.8 ± 185.6	240.1 ± 230.7	0.672
Median (range)	198 (5–834)	219 (5–1019)	0.959
ART regimen, n (%)			
PI-based	9 (18.4)	11 (24.5)	0.615
NNRTI-based	21 (42.8)	24 (53.3)	0.409
INSTI-based	19 (38.8)	10 (22.2)	0.117

*SD* standard deviation, *MSM* men who have sex with men, *PI* protease inhibitor, *INSTI* integrase strand transfer inhibitor, *NNRTI* non-nucleoside reverse transcriptase inhibitor, *ART* antiretroviral therapy

### Intervention effects on retention in HIV care

**Fig. 2:**
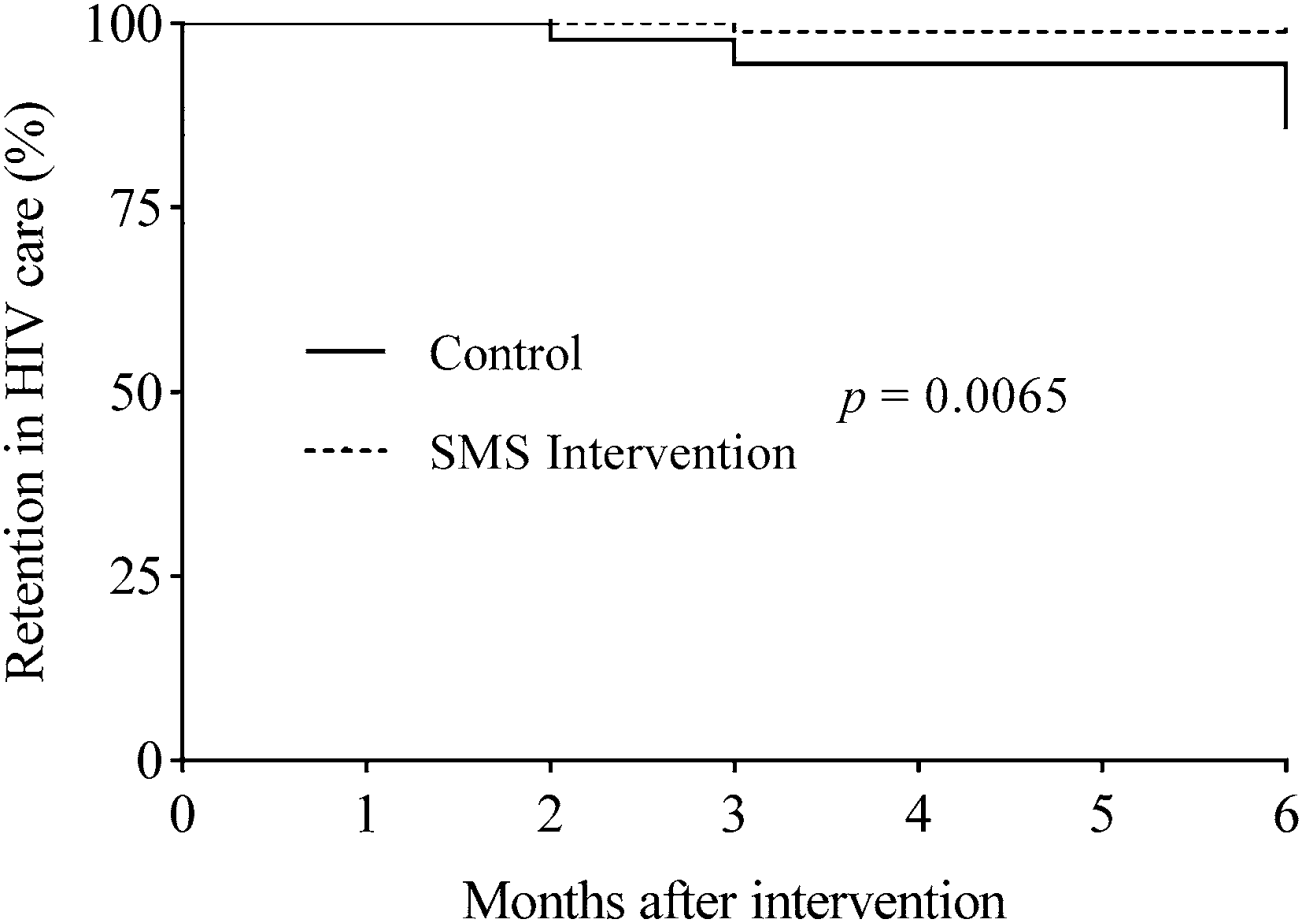
Effect of text messaging reminder on retention in HIV care. A Kaplan–Meier plot for two groups associated with patient retention over time

Across the two assessment points, 43 of 49 (88%) and 39 of 49 (80%) participants completed all follow-up visits at 3 and 6 months in the control group, and 44 of 45 (98%) and 44 of 45 (98%) in the intervention group, respectively (Table 2 and Fig. 2). Retention in care significantly improved in the intervention group compared to the control group with the odds of retention increasing by 20% (OR, 1.2; 95% CI 1.1–1.5, *p* = 0.006) at the end of the study period (Table 2), indicating that patient drop off occurred mostly in the control group, and receipt of the SMS was associated with a significant improvement in patient retention in medical care.

**Table 2:** Retention in care between intervention and control groups

	SMS group	Control group	OR (95% CI)	*p* value
Retention, n (%)				
At 8 weeks	45 (100)	46 (93.9)	1.1 (0.8–1.0)	0.092
At 3 months	44 (97.8)	43 (87.8)	1.1 (0.9–1.2)	0.065
At 6 months	44 (97.8)	39 (79.6)	1.2 (1.1–1.5)	0.006

*SMS* short message service, *OR* odds ratio, *CI* confidence interval

### Intervention effects on self-reported ART adherence

By an intent-to-treat analysis, the proportion of participants with a VAS of at least 90% was not significantly different between the two groups at 3-month (84.4% vs 77.6%, p = 0.396) and 6-month (86.7% vs 73.5%, p = 0.194) follow-up visits (Table 3, left panel). Additionally, the proportion of participants with a VAS adherence ≥ 90% in the intervention group increased from 84.6% at 3-month to 86.7% at 6-month follow-up (*p* = 0.764); comparable rates in the control group were 77.6% at 3-month and 73.5% at 6-month follow-up (*p* = 0.638).

**Table 3:** ART adherence at 3- and 6-month post-intervention

Adherence	Intent-to-treat				Per protocol			
	SMS group	Control group	OR (95% CI)	*p* value	SMS group	Control group	OR (95% CI)	*p* value
3 months								
VAS ≥ 90, n (%)	38 (84.4)	38 (77.6)	1.6 (0.6–4.1)	0.396	38 (86.4)	38 (88.4)	0.9 (0.8–1.2)	0.778
6 months								
VAS ≥ 90, n (%)	38 (84.4)	36 (73.5)	1.9 (0.7–5.0)	0.194	39 (88.6)	36 (92.3)	0.9 (0.8–1.1)	0.572

*SD* standard deviation, *ART* antiretroviral therapy, *SMS* short message service, *VAS* visual analogue scale, *OR* odds ratio, *CI* confidence interval

Similarly, among participants who were retained in care at 3-month and 6-month follow-up, there was no significant difference observed in the percent of patients with VAS adherence ≥ 90% between the two groups in either the 3-month (86.4% vs 88.4%, *p* = 0.778) or the 6-month (88.6% vs 92.3%, *p* = 0.572) follow-up by per-protocol analysis (Table 3, right panel). Also, there was no significant difference in the percent of patients with VAS adherence ≥ 90% from 3- to 6-month follow-up in the intervention group (86.4% vs 88.6%, *p* = 0.747) nor the control group (88.4% vs 92.3%, *p* = 0.549) by per-protocol analysis. However, lower retention in the control group may have impacted the adherence estimate in both intent-to-treat and per-protocol analyses.

### Intervention effects on biologic indicators (HIV RNA and CD4 cell counts)

**Fig. 3:**
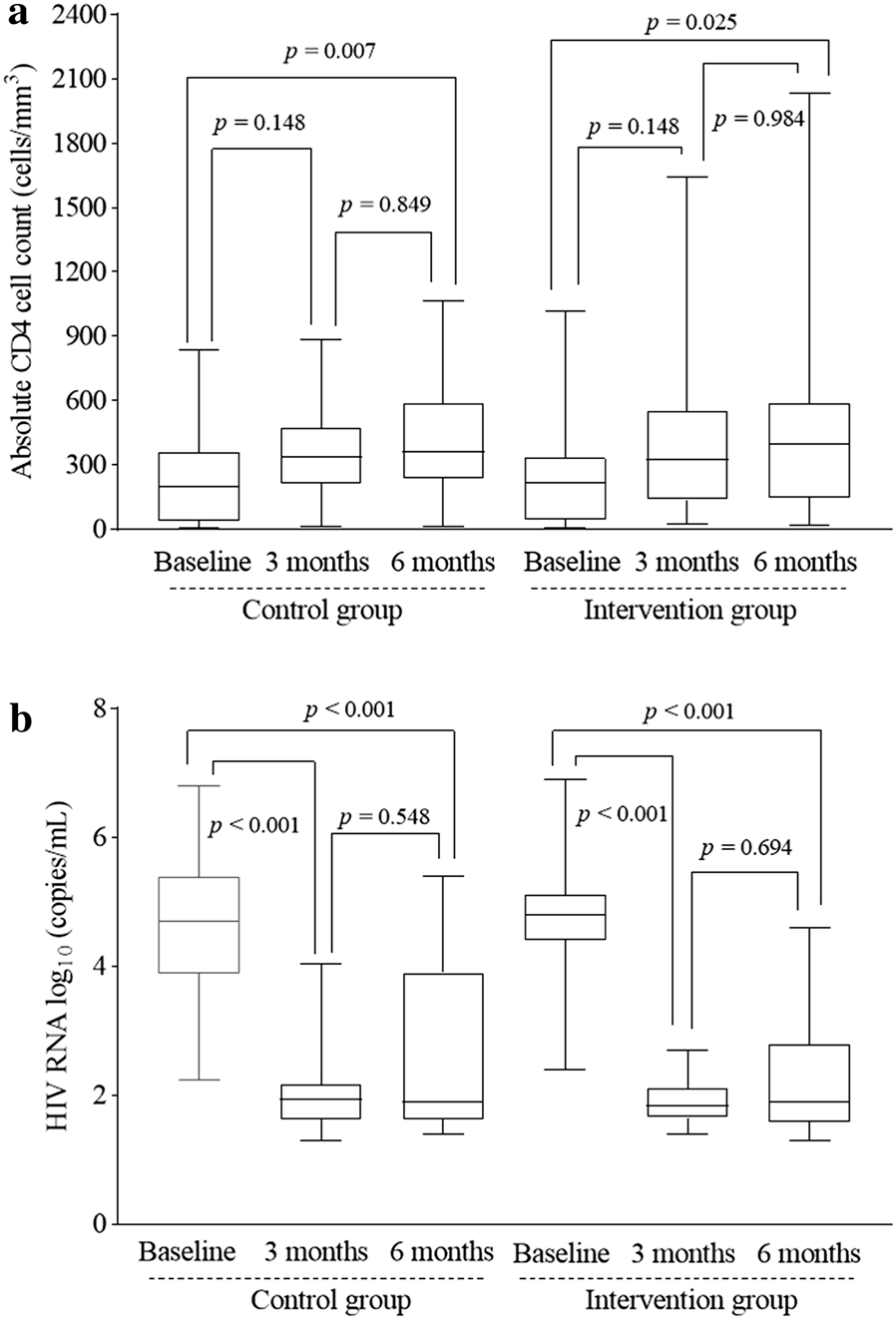
Intervention effects on HIV RNA load and CD4 cell count. Boxplots of mean CD4 cell count with range (**a**) and HIV RNA (log_10_ copies/mL) with range (**b**) in the control compared to the intervention group at baseline, 3 months and 6 months post-intervention

As shown in Table 4, there was no significant difference in term of CD4 cell counts, HIV RNA levels and the percent of patients with undetectable HIV RNA (< 50 copies/mL) between the two groups at either baseline, 3-month or 6-month follow-up. CD4 cell counts increased slightly from 240.1 ± 230.7 cells/µL at baseline to 357.9 ± 212.2 cells/µL (*p* = 0.148) at 3-month follow-up and increased significantly to 406.8 ± 254.4 cells/µL (*p* = 0.025) at 6-month follow-up in the intervention group; comparable increases in CD4 cell counts in the control group were 221 ± 185.6 cells/µL at baseline to 376.4 ± 300.1 cells/µL (*p* = 0.148) at 3-month follow-up and 413.2 ± 361.3 cells/µL (*p* = 0.007) at 6-month follow-up, however no difference was observed between 3-month and 6-month follow-up in either the control group (*p* = 0.849) or the intervention group (*p* = 0.984) (Fig. 3a). Geometric mean HIV RNA significantly decreased from 4.7 ± 0.8 (log_10_, copies/mL) at baseline to 2.0 ± 0.6 (log_10_, copies/mL) (*p* < 0.001) at 3-month follow-up and 2.5 ± 1.4 (log_10_, copies/mL) (*p* < 0.001) at 6-month follow-up in the intervention group; comparable decrease in HIV RNA in the control group were 4.7 ± 1.0 (log_10_, copies/mL) at baseline and 2.3 ± 1.0 (log_10_, copies/mL) (*p* < 0.001) and 2.2 ± 0.9 (log_10_, copies/mL) (*p* < 0.001) at 3- and 6-month follow-up visits respectively, whereas there was no significant difference in HIV RNA between 3-month and 6-month follow-up visits in either the control group (*p* = 0.548) or the intervention group (*p* = 0.694). Over the 6-month intervention period, the percent of participants with undetectable HIV RNA increased significantly from 68.2% at 3-month follow-up to 88.6% (*p* = 0.019) at 6-month follow-up in the intervention group; comparable rates in the control group were 69.5% at 3-month follow-up and 92.3% (*p* = 0.010) at 6-month follow-up visits.

**Table 4:** Intervention effects on CD4 cell counts and HIV RNA load

	SMS group	Control group	*p* value
CD4 cell counts, mean ± SD			
Baseline	221.8 ± 185.6	240.1 ± 230.7	0.672
At 3 months	376.4 ± 300.1	357.9 ± 212.2	0.765
At 6 months	415.2 ± 361.3	406.8 ± 254.4	0.902
HIV RNA, log, mean ± SD			
Baseline	4.7 ± 0.8	4.7 ± 1.0	1.000
At 3 months	2.0 ± 0.6	2.3 ± 1.0	0.095
At 6 months	2.5 ± 1.4	2.2 ± 0.9	0.244
Undetectable HIV RNA, n (%)			
Baseline	0 (0)	0 (0)	1.000
At 3 months	30 (66.7)	30 (61.2)	0.583
At 6 months	39 (86.7)	36 (73.5)	0.112

*SMS* short message service, *SD* standard deviation, *RNA* ribonucleic acid

## Discussion

Underinsured and uninsured PWH in the US are able to access HIV medical care and treatment via the RWHAP. In this pilot trial, we investigated the impact of a one-way SMS reminder intervention on ART adherence and retention in care for this group of patients. Over the 6-month study period, participants in the intervention group showed comparable ART adherence to those in the control group, with overall 86.7% versus 73.5% of participants with ≥ 90% adherence at study end (OR 2.0, 95% CI 0.7–5.0, *p* = 0.194). However, retention in care in the intervention group was higher than that seen in the control group, with the odds of retention increasing by 23% at intervention end (OR 1.2, 95% CI 1.1–1.5, *p* = 0.006). As expected, participants in both groups showed evidence of improved measures of HIV care (CD4 cell counts and HIV RNA load) following ART initiation. These results demonstrate that a one-way daily SMS reminder could provide substantial health benefits to RWHAP-supported PWH by improving retention in care. Retention in HIV care remains a critical public health imperative as those individuals who dropout from care are responsible for 61.3% of all HIV transmissions [25].

Despite efforts to promote ART adherence and retain PWH in medical care, half of known persons with HIV in the US are not engaged in regular care and only slightly more than half have achieved virologic suppression [4]. A recent US trial among adolescents and young adults found that self-reported ART adherence was higher among those who received personalized daily text messages [19]. Further, a meta-analysis of 1166 participants found that the impact of text messaging on ART adherence is influenced by level of education, gender, timing (weekly vs daily) and interactivity [26]. Our study is unique in that it is the first study to report the effect of a SMS reminder intervention on ART adherence specifically among low-income PWH receiving RWHAP-funded services. The population came from a variety of backgrounds with the majority being male, Black, behaviorally-infected and of low socioeconomic status. This population is often associated with poor ART adherence [27–29]. We previously published qualitative results demonstrating that patients in the intervention group felt the SMS reminders helped them with medication adherence [30]. As demonstrated in this analysis of quantitative results, daily SMS reminders did not affect ART adherence, but improved retention in medical care. We found that similar numbers of participants in the intervention group and the control group reported ≥ 90% adherence at 3- and 6-months post-intervention. However, 98% of participants in the intervention group were retained in care at 6 months post-intervention versus 80% in the control group, with the odds of retention increasing by 20% (OR, 1.2; 95% CI 1.1–1.5; *p* = 0.006). This is an important finding of our study, as our data demonstrate that retention in HIV care may be improved in PWH receiving RWHAP-funded services. Regarding efficacy, ART initiation was clearly associated with improvement in CD4 cell count in the 6 months of treatment (p < 0.001). The significant reduction in HIV RNA was noted in the first 3 months of treatment (p < 0.001) and there was a general plateau of HIV RNA reduction beyond 3 months of treatment in both groups.

In this study, the proportion of participants achieving adherence of at least 90% over the 6-month study period was comparable between the two groups. This might be because all participants enrolled in this study were newly initiating ART and were more cautious of their HIV self-management. Another plausible explanation is that our clinic receives RWHAP funding and, through this funding, offers a comprehensive set of services including a multidisciplinary health care team and social services personnel. As previously reported, virologic suppression rates are higher in RWHAP settings, likely due to the comprehensive care services offered to the patient [31]. Therefore, it may be that a SMS intervention cannot boost an already high adherence rate any further. Also, the lower retention in the control group may have impacted the adherence estimate analysis.

There are a few limitations in our study. Firstly, this was a single-center experience with US patients in the RWHAP. We cannot state with certainty that findings from this small sample can be generalized to wider populations of PHIV, including privately insured individuals. Secondly, the patients enrolled in this study were HIV treatment-naïve, newly initiated on ART and most received first-line treatment with a nonnucleoside reverse transcriptase inhibitor, reflective of the study recruitment period and contemporaneous US guideline-based treatment recommendations. We did not explore the impact of a SMS intervention on those with ART treatment experience and have limited data on contemporary ART regimens that are largely integrase-based. Lastly, the study only followed patients for 6 months and the results are thereby limited to short-term follow-up. Despite these limitations, given that engagement and retention in HIV care is an ongoing challenge, our finding that daily SMS reminders improved retention in care provides valuable insights to reduce the risk of secondary transmission and improve the management of HIV infection.

In summary, our study showed that 1-way daily SMS reminders over 6-months did not significantly improve ART adherence in HIV care among patients in a RWHAP clinic in south Florida, but significantly enhanced retention in early HIV care in this population. Larger controlled studies with long-term follow up are needed to determine the potential benefit of this technology-based intervention not only to improve retention in HIV care, but also to further explore the impact on ART adherence for PWH in lower-resource settings.

## Data Availability

The datasets used and/or analyzed during the current study are available from the corresponding author on reasonable request.
